# Medical students and interns’ attitude towards psychiatry as a postgraduate program in Saudi Arabia: A cross-sectional study

**DOI:** 10.1097/MD.0000000000046810

**Published:** 2025-12-26

**Authors:** Moayyad Alsalem, Mohammad Binyamin, Abdullah Kattan, Shuruq Alghamdi, Fares Sindi, Ahmed Alsaleh

**Affiliations:** a Psychiatry Section, Department of Medicine, King Abdulaziz Medical City, Ministry of the National Guard – Health Affairs, Jeddah, Saudi Arabia; b Department of Psychiatry, King Abdullah International Medical Research Center, Jeddah, Saudi Arabia; c College of Medicine, King Saud Bin Abdulaziz, University for Health Sciences, Jeddah, Saudi Arabia; d King Fahad Armed Forces Hospital, Jeddah, Saudi Arabia; e Erada Mental Health Complex, Jeddah, Saudi Arabia.

**Keywords:** attitude, medical, psychiatry, Saudi Arabia, students

## Abstract

Considering the intricate reasons behind a student’s choice of specialty, understanding these factors may help improve strategies used to recruit and direct students to psychiatry. Therefore, this study aims to examine the attitude of medical students towards psychiatry as a profession and its associated predictors. A cross-sectional online survey study was conducted in Saudi Arabia June 2023 and July 2024. This study utilized previously developed questionnaire tool, attitude towards psychiatry-30 scale by Burra et al. The attitude towards psychiatry-30 scale is a 30-item questionnaire for measuring the attitudes of medical. The predictors of having positive attitude towards psychiatry were identified using logistic regression. A total of 431 students participated in this study. The mean attitude score for the study participants was 94.0 (standard deviation: 10.7), out of 150 (equal to 62.7%), which reflects moderate attitude towards psychiatry as medical profession. The most commonly agreed upon statements were that “Psychiatric illness deserves at least as much attention as physical illness” and “It is interesting to try to unravel the cause of a psychiatric illness” with a mean score of 4.2 (1.1) and 4.2 (1.0), respectively. Binary logistic regression analysis identified that medical students’ sociodemographic characteristics did not affect their attitude towards psychiatry as medical profession (*P* > .05). The attitude of medical students in Saudi Arabia towards psychiatry as a medical profession was moderately positive. Policymakers need to work on the optimum inclusion of psychiatry in the curriculum, exposure to psychiatric rotations, and mentorship opportunities with practicing psychiatrists. Future research needs to develop targeted interventions to enhance positive attitudes and probably convince more students to consider psychiatry as a preferred choice.

## 1. Introduction

Psychiatry is a medical field that focuses on diagnosing, treating, and preventing mental illnesses, which have high diagnostic significance.^[1]^ Behavioral, emotional, and cognitive dysfunction are manifestations of mental illnesses.^[2]^ Mental illnesses include addictive behaviors, eating disorders, personality disorders, posttraumatic stress disorder, panic disorder, schizophrenia, bipolar disorder, and depression.^[3]^ It is now well established that mental illnesses are the primary reason for disease burden.^[4]^

Worldwide, approximately 970 million individuals suffered from mental illness in 2019, according to the World Health Organization.^[5]^ Likewise, in Saudi Arabia, the prevalence of mental illness at primary healthcare organizations is high, ranging from 30% to 46%.^[6]^ Mental illness can escalate to more severe consequences, including disability. Mental illness is reported to be associated with significant disability.^[7]^ Although there is a considerable burden and disability related to mental illness, psychiatry encounters one of the most extreme shortages compared to other medical specialties.^[8]^ Alerts have been raised about the consequential deficiency of available psychiatrists to cope with the burden of mental illness, severely limiting the care of this vulnerable group of mentally ill patients.^[9]^

Commonly identified reasons for the lack of interest in psychiatry include as a career are negative comments from family and friends for specializing in psychiatry, insufficient income, and relatively low respect compared with other specialties of medicine.^[10,11]^ Previous research has shown that multiple factors influence medical students’ choice of psychiatry as a specialty, including lifestyle, income, cultural factors, and availability of jobs.^[12–15]^ Still, the vital factor that determines the decision of medical students about their readiness to treat psychiatric conditions in clinical settings and to choose psychiatry as a profession is their attitudes toward mental illness and psychiatry.^[16]^

Considering the intricate reasons behind a student’s choice of specialty, by identifying these factors, it is possible to develop strategies that can be used to recruit and direct students to psychiatry.^[17]^ Several studies have explored medical students’ attitudes toward psychiatry and their career intentions to understand the factors that affect their choices more reasonably.^[13,18–28]^ Despite the high prevalence of mental illness in Saudi Arabia, there is deficient research in this area. Therefore, this study aims to examine the attitude of medical students towards psychiatry as a profession and its associated predictors.

## 2. Methods

### 2.1. Study design

A cross-sectional online survey study was conducted in Saudi Arabia June 2023 and July 2024 to examine the attitude of medical students towards psychiatry as a profession and its associated predictors.

### 2.2. Sampling strategy and study population

The eligible participants were those identified using the convenient sampling technique, and then they were invited to take part in this study. Informed consents were taken through social media platforms, including Facebook, WhatsApp, Twitter, and Instagram. We explained the aims and objectives of the study in detail at the cover letter of the survey. Medical students and interns of different colleges (males and females’ medical interns and students in the any of the 3 phases: foundation, preclinical, and clinical years, respectively) in Saudi Arabia formed the study population. All undergraduates and interns in colleges of medicine and surgery were included. Undergraduate and postgraduate candidates from either the nursing college, dental college, or pharmacy college were excluded.

### 2.3. Study tool

This study utilized previously developed questionnaire tool, attitude towards psychiatry-30 scale by Burra et al.^[29]^ The attitude towards psychiatry-30 scale is a 30-item questionnaire for measuring the attitudes of medical students, health professionals, or the general public towards psychiatry. The scale has been constructed for measuring attitude with respect to the profession of psychiatric medicine and understanding how such attitudes might relate to career choices, treatment approaches, and perceptions of mental health care. Statements related to psychiatry rated by respondents on a 5-point Likert scale ranging from “Strongly Disagree” to “Strongly Agree.” The scale is balanced with both positively and negatively worded items; the latter are reverse scored. The total score is between 30 and 150, with higher scores indicating more positive attitudes toward psychiatry.^[29]^ In addition, we collected demographic data related to participants’ gender, age, nationality, year of study, and source of information regarding the field of psychiatry.

### 2.4. Ethical approval

This study was approved by the Institutional Review Board (IRB) King Abdullah International Medical Research Center, Jeddah, Saudi Arabia (IRB/1293/23).

### 2.5. Statistical analysis

All statistical analyses were done using SPSS, version 29. Descriptive statistics were used to describe the demographic features of participants in the study. Continuous data for normally distributed variables were presented as mean ± standard deviation (SD). The categorical data were presented as percentages, with frequencies. Odds ratio and 95% confidence intervals for the predictors of having positive attitude (participants who have score equal to or above the mean score for the study sample, which is 94.0) towards psychiatry were identified using logistic regression. The cutoff for logistic regression was based on the mean score of the study participants (94.0 [SD: 10.7]).

## 3. Results

A total of 431 students participated in this study. More than half of them (57.1%) were males. Around half of them (49.2%) were aged 21 to 23 years. The vast majority of them (94.4%) were Saudis. Around one-quarter of them (23.2%) were at their fifth year of study. College curriculum (83.5%) was the most commonly reported source of information on psychiatry. For further details on students’ demographic characteristics, refer to Table 1.

**Table 1:** Students’ demographic characteristics.

Variable	Frequency	Percentage
Gender		
Male	246	57.1%
Age group		
18–20 yr	164	38.1%
21–23 yr	212	49.2%
24–26 yr	39	11.4%
27–29 yr	3	0.7%
30–32 yr	3	0.7%
Nationality		
Saudi	407	94.4%
Year of study		
First year	69	16.0%
Second year	85	19.7%
Third year	30	7.0%
Fourth year	88	20.4%
Fifth year	100	23.2%
Sixth year	23	5.3%
Intern	36	8.4%
Source of information regarding the field of psychiatry (multiple answers question)		
College curriculum	360	83.5%
Social media	49	11.4%
Books/journals	17	3.9%
Extracurricular activities	1	0.2%
Relative is a psychiatrist	1	0.2%
Psychiatry consultant	1	0.2%
Family members	1	0.2%
Google	1	0.2%
Senior experiences	1	0.2%
All of the above	2	0.5%

### 3.1. Attitudes towards psychiatry

The mean attitude score for the study participants was 94.0 (SD: 10.7), out of 150 (equal to 62.7%), which reflects moderate attitude towards psychiatry as medical profession. The most commonly agreed upon statements were that “Psychiatric illness deserves at least as much attention as physical illness” and “It is interesting to try to unravel the cause of a psychiatric illness” with a mean score of 4.2 (1.1) and 4.2 (1.0), respectively. The least commonly agreed upon statement was that “Psychiatrists seem to talk about nothing but sex” with a mean score of 2.0 (1.0), Table 2.

**Table 2:** Participants’ mean attitude score.

Attitude item	Mean (standard deviation)
Psychiatry is unappealing because it makes so little use of medical training	2.5 (1.3)
Psychiatrist talk a lot but do very little	2.7 (1.2)
Psychiatric hospitals are little more than prisons	2.9 (1.2)
I would like to be a psychiatrist	2.5 (1.3)
It is quite easy for me to accept the efficacy of psychotherapy	3.6 (1.1)
On the whole people taking up psychiatric training are running away from participation in real medicine	2.3 (1.1)
Psychiatrists seem to talk about nothing but sex	2.0 (1.0)
The practice of psychotherapy basically is fraudulent since there is no strong evidence that it is effective	2.1 (1.1)
Psychiatric teaching increases our understanding of medical and surgical patients	3.5 (1.2)
The majority of students report that their psychiatric undergraduate training has been valuable	3.3 (1.0)
Psychiatry is a respected branch of medicine	3.9 (1.1)
Psychiatric illness deserves at least as much attention as physical illness	4.2 (1.1)
Psychiatry has very little scientific information to go on	2.8 (1.3)
With the forms of therapy now at hand most psychiatric patients improve	3.7 (1.0)
Psychiatrists tend to be at least as stable as the average doctor	3.2 (1.1)
Psychiatric treatment causes patients to worry too much about their symptoms	3.0 (1.1)
Psychiatrists get less satisfaction from their work than other specialists	2.9 (1.1)
It is interesting to try to unravel the cause of a psychiatric illness	4.2 (1.0)
There is very little that psychiatrists can do for their patients	2.5 (1.3)
Psychiatric hospitals have a specific contribution to make to the treatment of the mentally ill	3.8 (1.0)
If I were asked what I considered to be the 3 most exciting medical specialties, psychiatry would be excluded	3.2 (1.5)
At times it is hard to think of psychiatrists as equal to other doctors	2.6 (1.3)
These days psychiatry is the most important part of the curriculum in medical schools	3.3 (1.3)
Psychiatry is so unscientific that even psychiatrists can’t as to what its basic applied sciences are	2.6 (1.1)
In recent years psychiatric treatment has become quite effective	3.9 (1.0)
Most of the so-called facts in psychiatry are really just vague speculations	2.6 (1.1)
If we listen to them, psychiatric patients are just as human as other people	4.1 (1.1)
The practice of psychiatry allows the development of really rewarding relationships with people	3.9 (1.0)
Psychiatric patients are often more interesting to work with than other patients	3.2 (1.2)
Psychiatry is so amorphous that it cannot really be taught effectively	3.0 (1.1)

### 3.2. Predictors of positive attitude towards psychiatry

Binary logistic regression analysis identified that medical students’ sociodemographic characteristics did not affect their attitude towards psychiatry as medical profession (*P* > .05), Table 3.

**Table 3:** Predictors of positive attitude towards psychiatry.

Variable	Odds ratio (95% confidence interval)	*P*-value
Gender		
Female (reference category)	1.00	
Male	0.89 (0.61–1.31)	.557
Age group		
18–20 yr (reference category)	1.00	
21–23 yr	1.12 (0.74–1.69)	.589
24–26 yr	1.04 (0.55–1.98)	.902
27–29 yr	2.56 (0.23–28.75)	.447
Nationality		
Non-Saudi (reference category)	1.00	
Saudi	1.75 (0.73–4.18)	.207
Year of study		
First year (reference category)	1.00	
Second year	0.81 (0.43–1.54)	.518
Third year	1.16 (0.49–2.73)	.740
Fourth year	1.39 (0.74–2.61)	.310
Fifth year	0.71 (0.38–1.32)	.278
Sixth year	0.74 (0.28–1.95)	.546
Intern	1.62 (0.72–3.65)	.246

## 4. Discussion

The current investigation found that the most commonly reported source of information on psychiatry was college curriculum (83.5%). In addition, the mean attitude score for the study participants was 94.0 (SD: 10.7), out of 150 (equal to 62.7%), which reflects a moderate attitude towards psychiatry as a medical profession. These findings align with prior studies that underscore the significance of education curricula in raising knowledge and shaping medical students’ attitudes about psychiatry.

In line with our findings about the college curriculum as a primary source of psychiatry information, a prior study in Uganda showed that introducing psychiatry into the curriculum at the beginning of the fourth year of medical school is associated with higher levels of knowledge of psychiatry for a fourth-year student.^[30]^ Likewise, prior research from Canada,^[31]^ Burkina Faso,^[32]^ India,^[33]^ and Indonesia^[34]^ revealed that medical students at advanced years of study and age have better knowledge of psychiatry. An earlier study in Jeddah also concluded that the undergraduate academic curriculum in psychiatry can enhance students’ attitudes toward mentally ill people by incorporating strategies to destigmatize psychiatric and mental illnesses people into the curriculum.^[35]^ These results emphasize the important role of the university academic curriculum in providing students with knowledge about psychiatry and improving their attitudes toward it.

The moderate attitude towards psychiatry as a medical profession indicates that improvement is needed. In these terms, a previous study in Riyadh found that exposure to psychiatry during medical school was limited, leading to a significant proportion of students having a negative perspective of the specialty.^[36]^ In that earlier study, students identified factors that led to their unwillingness to specialize in psychiatry as lack of adequate exposure to specialty and ward experiences, perceived disdain for the role of psychiatrists by the general community (nonmedical), and lack of immediate gratification when treating mentally ill patients.^[36]^ To overcome these challenges, a prior study conducted in the Al-Hassa Medical College concluded many recommendations, which include the need to treat students with positive attitudes towards psychiatry during their training period, enhance psychiatry education curricula, and focus programs on improving positive communication between medical students and patients suffering from mental disorders.^[37]^ Similarly, an earlier study conducted in India reported that the attitudes of students toward psychiatry were negative and revealed that modifying the medical curriculum can improve students’ attitudes toward psychiatry.^[38]^ In addition to curricula, a previous study found other sources for psychiatry knowledge among medical students, like research and social media.^[30]^ Thus, using multiple psychiatry sources could improve the medical students’ understanding of and attitudes toward psychiatry.

Indeed, several studies have found positive effects of specific education and training programs and completion of university training on individuals’ attitudes toward mental illness and psychiatry.^[39–42]^ Besides, implementing a supplementary educational program may help improve students’ attitudes toward psychiatry. In Japan, implementing such programs for 60 minutes in addition to the curriculum improved students’ attitudes significantly.^[43,44]^ On the other hand, in Tunisia, students completed psychiatric training but did not achieve an improvement in the level of stigma toward patients with mental illness.^[45]^ The Tunisian study demonstrated the requirement to enhance curricula to obtain more positive attitudes.^[45]^

To enhance young academics’ recruitment into psychiatry, psychological organizations have undertaken initiatives to understand better their hesitations towards the field.^[46–49]^ More research is needed to understand the reasons for the low attitude towards psychiatry in the region and ensure that curricula are updated to meet current requirements and provide students with the necessary knowledge and thus improve their attitude towards psychiatry.

The current investigation found that the most commonly agreed upon statements regarding attitude were that “Psychiatric illness deserves at least as much attention as physical illness” and “It is interesting to try to unravel the cause of a psychiatric illness” with a mean score of 4.2 (1.1) and 4.2 (1.0), respectively. These findings are consistent with previous studies highlighted that most of their participants felt that “Psychiatric illness deserves at least as much attention as physical illness”^[50,51]^ and that “It is interesting to try to unravel the cause of a psychiatric illness.”^[50]^ These findings indicate positive attitudes towards psychiatry. The recognition that mental illnesses deserve at least as much attention as physical illnesses has shown a growing awareness and understanding of mental illness among medical students, as mental health is a vital part of a person’s functioning and well-being.^[52]^ In addition, students’ interest in discovering the cause of mental illness means that they will research the illness, and their knowledge and attitude will improve because research contributes to improving students’ attitudes about mental illnesses, as we mentioned previously. Hence, using targeted educational strategies and providing research opportunities for students may contribute to the continued growth of positive attitudes towards psychiatry and thus recruit enough students to specialize in this field.

Another important finding of our study is that the least commonly agreed upon statement regarding attitude was that “Psychiatrists seem to talk about nothing but sex” with a mean score of 2.0 (1.0). This finding reflects increased understanding and awareness of psychiatry and may attributed to improved education. However, in both developed and developing countries, there are persistent cultural stereotypes associated with psychiatry as a specialty, psychiatric patients, and psychiatrists.^[53]^ Thus, any remaining cultural stereotypes or stigma must continue to be addressed to ensure that negative student attitudes about psychiatry are not built up and thus continue to build more positive attitudes and attract more students to the specialty.

In our study, binary logistic regression analysis identified that medical students’ sociodemographic characteristics did not affect their attitude toward psychiatry as the medical profession. This indicates that individual experiences and educational environment shape attitudes for students about physiatry more than demographic factors. Thus, educational interventions aimed at enhancing attitudes toward psychiatry can be applied across all demographic groups, making it feasible to implement constant improvements in psychiatric education that benefit all students.

This study has limitations. The ability to examine causality across the study variables is limited due to the cross-sectional study design. The use of online survey study design has limited generalizability as the study population is restricted to users of social media platforms (e.g., 94.4% were Saudis). The lack of qualitative data that provide in-depth understanding of medical students and interns’ attitude towards psychiatry as a postgraduate program is another limitation. Besides, self-administered studies are prone to reporting bias and social desirability bias. Therefore, the study findings should be interpreted carefully. Future qualitative studies are recommended to examine nuanced perceptions concerning medical students’ attitude towards psychiatry as a postgraduate program. Qualitative research has the advantage of providing deeper understanding of students’ perspectives and more contextual insights.

## 5. Conclusion

Medical students in Saudi Arabia demonstrated moderate attitude towards psychiatry as medical profession. Efforts by policymakers should focus on making optimal inclusion of psychiatry in the curriculum by providing exposure to psychiatric rotations, including more experiential components in psychiatry training, and mentorship opportunities with practicing psychiatrists. Future research should aim for the development of targeted interventions to enhance positive attitudes and probably convince more students to consider psychiatry as a preferred choice.

## Author contributions

**Conceptualization:** Moayyad Alsalem, Mohammad Binyamin, Ahmed Alsaleh.

**Data curation:** Moayyad Alsalem, Mohammad Binyamin.

**Formal analysis:** Moayyad Alsalem, Mohammad Binyamin.

**Funding acquisition:** Moayyad Alsalem, Mohammad Binyamin.

**Investigation:** Moayyad Alsalem, Mohammad Binyamin, Abdullah Kattan, Shuruq Alghamdi, Fares Sindi, Ahmed Alsaleh.

**Methodology:** Moayyad Alsalem, Mohammad Binyamin.

**Project administration:** Moayyad Alsalem, Mohammad Binyamin.

**Resources:** Moayyad Alsalem, Mohammad Binyamin, Abdullah Kattan, Shuruq Alghamdi, Fares Sindi, Ahmed Alsaleh.

**Software:** Moayyad Alsalem, Mohammad Binyamin.

**Supervision:** Moayyad Alsalem, Ahmed Alsaleh.

**Validation:** Moayyad Alsalem, Mohammad Binyamin, Ahmed Alsaleh.

**Visualization:** Moayyad Alsalem, Mohammad Binyamin, Abdullah Kattan, Shuruq Alghamdi, Fares Sindi, Ahmed Alsaleh.

**Writing – original draft:** Moayyad Alsalem, Mohammad Binyamin, Abdullah Kattan, Shuruq Alghamdi, Fares Sindi, Ahmed Alsaleh.

**Writing – review & editing:** Moayyad Alsalem, Mohammad Binyamin, Abdullah Kattan, Shuruq Alghamdi, Fares Sindi, Ahmed Alsaleh.

## References

[1] Radaic A Martins-de-Souza D. The state of the art of nanopsychiatry for schizophrenia diagnostics and treatment. Nanomedicine. 2020;28:102222. 32439429 10.1016/j.nano.2020.102222

[2] Vargas T Damme KSF Ered A. Neuroimaging markers of resiliency in youth at clinical high risk for psychosis: a qualitative review. Biol Psychiatry Cogn Neurosci Neuroimaging. 2021;6:166–77. 32788085 10.1016/j.bpsc.2020.06.002 PMC7725930

[3] Doran C. Prescribing Mental Health Medication: The Practitioner’s Guide. 3rd ed. Routledge; 2021.

[4] Patel V Saxena S Lund C. The lancet commission on global mental health and sustainable development. Lancet. 2018;392:1553–98. 30314863 10.1016/S0140-6736(18)31612-X

[5] WHO. Mental health. https://www.who.int/health-topics/mental-health#tab=tab_2. Accessed August 16, 2024.

[6] Alghadeer SM Alhossan AM Al-Arifi MN. Prevalence of mental disorders among patients attending primary health care centers in the capital of Saudi Arabia. Neurosciences (Riyadh). 2018;23:239–43. 30008000 10.17712/nsj.2018.3.20180058 PMC8015574

[7] Chaudhury PK Deka K Chetia D. Disability associated with mental disorders. Indian J Psychiatry. 2006;48:95–101. 20703393 10.4103/0019-5545.31597 PMC2913573

[8] Darves B. Physician shortage spikes demand in several specialties. New England J Med. 2017;1:1.

[9] Weiner S. Addressing the Escalating Psychiatrist Shortage. AAMC News; 2018. https://www.aamc.org/news/addressing-escalating-psychiatrist-shortage.

[10] Wiesenfeld L Abbey S Takahashi SG Abrahams C. Choosing psychiatry as a career: motivators and deterrents at a critical decision-making juncture. Can J Psychiatry. 2014;59:450–4. 25161070 10.1177/070674371405900808 PMC4143302

[11] Balon R Franchini GR Freeman PS Hassenfeld IN Keshavan MS Yoder E. Medical students’ attitudes and views of psychiatry: 15 years later. Acad Psychiatry. 1999;23:30–6.

[12] AlOsaimi FMS AlShehri HM AlHasson WI Agha S Omair A. Why psychiatry as a career: effect of factors on medical students’ motivation. J Family Med Prim Care. 2019;8:648–51. 30984688 10.4103/jfmpc.jfmpc_399_18 PMC6436284

[13] Kuhnigk O Strebel B Schilauske J Jueptner M. Attitudes of medical students towards psychiatry: effects of training, courses in psychiatry, psychiatric experience and gender. Adv Health Sci Educ Theory Pract. 2007;12:87–101. 17115252 10.1007/s10459-005-5045-7

[14] Farooq K Lydall GJ Malik A. Why medical students choose psychiatry – a 20 country cross-sectional survey. BMC Med Educ. 2014;14:12. 24422951 10.1186/1472-6920-14-12 PMC3974144

[15] Malhi GS Coulston CM Parker GB. Who picks psychiatry? Perceptions, preferences and personality of medical students. Aust N Z J Psychiatry. 2011;45:861–70. 21923533 10.3109/00048674.2011.604301

[16] Al-Adawi S Dorvlo AS Bhaya C. Withering before the sowing? A survey of Oman’s “tomorrow’s doctors” interest in psychiatry. Educ Health (Abingdon). 2008;21:117. 19034834

[17] Sierles FS Yager J Weissman SH. Recruitment of U.S. medical graduates into psychiatry: reasons for optimism, sources of concern. Acad Psychiatry. 2003;27:252–9. 14754848 10.1176/appi.ap.27.4.252

[18] O’Connor K Brennan D O’loughlin K. Attitudes towards patients with mental illness in Irish medical students. Ir J Med Sci. 2013;182:679–85. 23605088 10.1007/s11845-013-0955-5

[19] Mortlock AM Puzzo I Taylor S. Enrichment activities in the medical school psychiatry programme – could this be a key to engaging medical students in psychiatry? A study from a high secure forensic psychiatric UK hospital. BMC Psychiatry. 2017;17:83. 28298188 10.1186/s12888-017-1236-z PMC5353898

[20] Aslam M Taj T Ali A. Psychiatry as a career: a survey of factors affecting students’ interest in psychiatry as a career. Mcgill J Med. 2009;12:7–12. 19753292 PMC2687919

[21] Halder N Hadjidemetriou C Pearson R. Student career choice in psychiatry: findings from 18 UK medical schools. Int Rev Psychiatry. 2013;25:438–44. 24032499 10.3109/09540261.2013.824414

[22] Baller FA Ludwig KV Kinas-Gnadt Olivares CL Graef-Calliess I-T. Exploring the ideas and expectations of German medical students towards career choices and the speciality of psychiatry. Int Rev Psychiatry. 2013;25:425–30. 24032497 10.3109/09540261.2013.823384

[23] Kuhnigk O Hofmann M Böthern AM Haufs C Bullinger M Harendza S. Influence of educational programs on attitudes of medical students towards psychiatry: effects of psychiatric experience, gender, and personality dimensions. Med Teach. 2009;31:e303–10. 19811138 10.1080/01421590802638048

[24] Zwerenz R Barthel Y Leuzinger-Bohleber M. Attitudes of medical students towards psychotherapeutic treatment and training. Z Psychosom Med Psychother. 2007;53:258–72. 17883933 10.13109/zptm.2007.53.3.258

[25] Strebel B Obladen M Lehmann E Gaebel W. Attitude of medical students to psychiatry. A study with the German translated, expanded version of the ATP-30. Nervenarzt. 2000;71:205–12. 10756529 10.1007/s001150050030

[26] Farooq K Lydall GJ Bhugra D. What attracts medical students towards psychiatry? A review of factors before and during medical school. Int Rev Psychiatry. 2013;25:371–7. 24032490 10.3109/09540261.2013.823855

[27] Sartorius N Gaebel W Cleveland H-R. WPA guidance on how to combat stigmatization of psychiatry and psychiatrists. World Psychiatry. 2010;9:131–44. 20975855 10.1002/j.2051-5545.2010.tb00296.x PMC2948719

[28] Lyons Z. Attitudes of medical students toward psychiatry and psychiatry as a career: a systematic review. Acad Psychiatry. 2013;37:150–7. 23632923 10.1176/appi.ap.11110204

[29] Burra P Kalin R Leichner P. The ATP 30 – a scale for measuring medical students’ attitudes to psychiatry. Med Educ. 1982;16:31–8. 7057722 10.1111/j.1365-2923.1982.tb01216.x

[30] Kihumuro RB Kaggwa MM Kintu TM. Knowledge, attitude and perceptions of medical students towards mental health in a university in Uganda. BMC Med Educ. 2022;22:730. 36266646 10.1186/s12909-022-03774-0 PMC9584261

[31] Riffel T Chen SP. Exploring the knowledge, attitudes, and behavioural responses of healthcare students towards mental illnesses – a qualitative study. Int J Environ Res Public Health. 2019;17:25. 31861420 10.3390/ijerph17010025 PMC6981946

[32] Sawadogo KCC Lameyre V Gerard D Bruand P-E Preux P-M. Knowledge, attitudes and practices in mental health of health professionals at the end of their curriculum in Burkina Faso: a pilot study. Nurs Open. 2020;7:589–95. 32089856 10.1002/nop2.427 PMC7024628

[33] Aruna G Mittal S Yadiyal MB Acharya C Acharya S Uppulari C. Perception, knowledge, and attitude toward mental disorders and psychiatry among medical undergraduates in Karnataka: a cross-sectional study. Indian J Psychiatry. 2016;58:70–6. 26985108 10.4103/0019-5545.174381 PMC4776586

[34] Puspitasari IM Garnisa IT Sinuraya RK Witriani W. Perceptions, knowledge, and attitude toward mental health disorders and their treatment among students in an Indonesian University. Psychol Res Behav Manag. 2020;13:845–54. 33149708 10.2147/PRBM.S274337 PMC7602896

[35] Kattan W Munshi I Alattas A Alsomali A Alghamdi H Alshehri W. Attitude toward psychiatry amongst medical students in Jeddah. Int J Med Dev Countries. 2022;6:1549–56.

[36] Ahmad M Dabbagh M Dabaliz A Obeidat A. Attitudes of medical students towards choosing psychiatry as a career. BJPsych Open. 2021;7:S2–3.

[37] El-Gilany AH Amr M Iqbal R. Students’ attitudes toward psychiatry at Al-Hassa Medical College, Saudi Arabia. Acad Psychiatry. 2010;34:71–4. 20071736 10.1176/appi.ap.34.1.71

[38] Yadav T Arya K Kataria D Balhara YPS. Impact of psychiatric education and training on attitude of medical students towards mentally ill: a comparative analysis. Ind Psychiatry J. 2012;21:22–31. 23766574 10.4103/0972-6748.110944 PMC3678174

[39] Altindag A Yanik M Ucok A Alptekin K Ozkan M. Effects of an antistigma program on medical students’ attitudes towards people with schizophrenia. Psychiatry Clin Neurosci. 2006;60:283–8. 16732743 10.1111/j.1440-1819.2006.01503.x

[40] Baxter H Singh SP Standen P Duggan C. The attitudes of “tomorrow’s doctors” towards mental illness and psychiatry: changes during the final undergraduate year. Med Educ. 2001;35:381–3. 11319003 10.1046/j.1365-2923.2001.00902.x

[41] Pinfold V Toulmin H Thornicroft G Huxley P Farmer P Graham T. Reducing psychiatric stigma and discrimination: evaluation of educational interventions in UK secondary schools. Br J Psychiatry. 2003;182:342–6. 12668411 10.1192/bjp.182.4.342

[42] Corrigan PW Green A Lundin R Kubiak MA Penn DL. Familiarity with and social distance from people who have serious mental illness. Psychiatr Serv. 2001;52:953–8. 11433114 10.1176/appi.ps.52.7.953

[43] Chew-Graham CA Rogers A Yassin N. “I wouldn’t want it on my CV or their records”: medical students’ experiences of help-seeking for mental health problems. Med Educ. 2003;37:873–80. 12974841 10.1046/j.1365-2923.2003.01627.x

[44] Mino Y Yasuda N Tsuda T Shimodera S. Effects of a one-hour educational program on medical students’ attitudes to mental illness. Psychiatry Clin Neurosci. 2001;55:501–7. 11555346 10.1046/j.1440-1819.2001.00896.x

[45] Brahmi L Amamou B Ben Haouala A Mhalla A Gaha L. Attitudes toward mental illness among medical students and impact of temperament. Int J Soc Psychiatry. 2022;68:1192–202. 35147058 10.1177/00207640221077551

[46] Schneider F Falkai P, Maier W. Psychiatrie 2020 Plus: Perspektiven, Chancen und Herausforderungen. 2nd ed. Springer; 2012. https://link.springer.com/book/10.1007/978-3-642-28221-8.

[47] European Confederation, S. eGovernment Strategy 2017–2019. 2016. https://eur-lex.europa.eu/legal-content/en/TXT/?uri=CELEX%3A52016DC0179.

[48] Möller-Leimkühler AM Möller H-J Maier W Gaebel W Falkai P. EPA guidance on improving the image of psychiatry. Eur Arch Psychiatry Clin Neurosci. 2016;266:139–54. 26874959 10.1007/s00406-016-0678-5

[49] WHO. mhGAP: Mental Health Gap Action Programme: Scaling up Care for Mental, Neurological and Substance Use Disorders. World Health Organization; 2008. https://www.who.int/publications/i/item/9789241596206. 26290926

[50] Patra S Patro BK. What they think of us: a study of teaching medical specialists’ attitude towards psychiatry in India. Indian J Psychiatry. 2017;59:100–5. 28529368 10.4103/0019-5545.204434 PMC5418994

[51] Patra S Patro BK Nebhinani N. Images of psychiatry: attitude survey of teaching medical specialists of India. Ind Psychiatry J. 2017;26:52–5. 29456322 10.4103/ipj.ipj_36_16 PMC5810168

[52] Gautam S Jain A Chaudhary J Gautam M Gaur M Grover S. Concept of mental health and mental well-being, it’s determinants and coping strategies. Indian J Psychiatry. 2024;66:S231–44. 38445271 10.4103/indianjpsychiatry.indianjpsychiatry_707_23 PMC10911315

[53] Jilowa CS Meena PS Jain M. Attitude of undergraduate medical students toward psychiatry: a cross-sectional comparative study. Ind Psychiatry J. 2018;27:124–30. 30416303 10.4103/ipj.ipj_82_17 PMC6198587

